# The Stability Maintenance of Protein Drugs in Organic Coatings Based on Nanogels

**DOI:** 10.3390/pharmaceutics12020115

**Published:** 2020-02-01

**Authors:** Hongzhao Qi, Lijun Yang, Peipei Shan, Sujie Zhu, Han Ding, Sheng Xue, Yin Wang, Xubo Yuan, Peifeng Li

**Affiliations:** 1 Institute for Translational Medicine, Qingdao University, Qingdao 266021, China; 15000477094@126.com (P.S.); zhusujie87@126.com (S.Z.); dinghan2011@163.com (H.D.); shengxue198@126.com (S.X.); wangyin@sibs.ac.cn (Y.W.); peifli@hotmail.com (P.L.); 2 College of Materials Science and Engineering, Qingdao University of Science and Technology, Qingdao 266042, China; lijunyang@qust.edu.cn; 3 Tianjin Key Laboratory of Composite and Functional Materials, School of Materials Science and Engineering, Tianjin University, Tianjin 300072, China; xbyuan@tju.edu.cn

**Keywords:** protein drug, nanogel, organic solvent, organic coating, stability

## Abstract

Protein drugs are often loaded on scaffolds with organic coatings to realize a spatiotemporal controlled release. The stability or activity of protein drugs, however, is largely affected by the organic coating, particularly with organic solvents, which can dramatically reduce their delivery efficiency and limit their application scope. In spite of this, little attention has been paid to maintaining the stability of protein drugs in organic coatings, to date. Here, we used catalase as a model protein drug to exploit a kind of chemically cross-linked nanogel that can efficiently encapsulate protein drugs. The polymeric shells of nanogels can maintain the surface hydration shell to endow them with a protein protection ability against organic solvents. Furthermore, the protection efficiency of nanogels is higher when the polymeric shell is more hydrophilic. In addition, nanogels can be dispersed in polylactic acid (PLA) solution and subsequently coated on scaffolds to load catalase with high activity. To the best of our knowledge, this is the first use of hydrophilic nanogels as a protection niche to load protein drugs on scaffolds through an organic coating, potentially inspiring researchers to exploit new methods for protein drug loading.

## 1. Introduction

Protein drugs possessing high activity, high specificity, and low toxicity have been proven to be powerful therapeutics [1,2]. Most protein drugs, such as monoclonal antibodies [3], are currently used as injection formulations. However, the moderate bioavailability of protein drugs often results in their low concentration in the lesion after systemic administration [4]. Therefore, many organic or inorganic scaffolds are currently under investigation for the local controlled release of protein drugs [5,6]. To load proteins on these scaffolds, organic coatings are often adopted [7,8]. Researchers respectively dissolve proteins and polymers, such as polylactic acid (PLA), in the aqueous phase and an organic solvent to create emulsions. 

The protein-loaded organic coating can be formed on the scaffold after the extraction or evaporation of the organic solvent. This emulsion method can increase the loading amount of proteins and realize their controlled release through the degradation of polymers, enhancing the local concentration of protein drugs. For example, bone morphogenetic protein-2 (BMP-2) can be encapsulated in microspheres and distributed on the scaffolds to induce bone regeneration [9]. 

Despite some occasional success with this preparation process, significant problems remain with the stability of proteins. The fact that the water/organic solvent interface can denature proteins has been proven. Marco van de Weert et al. have reported that emulsification of lysozyme-containing aqueous solutions with methylene chloride causes incomplete protein recovery and the unrecovered lysozyme is observed at the oil-water interface as a white precipitate [10]. Hongkee Sah demonstrates that protein instability during emulsification is traced to the consequences of the adsorption and conformational rearrangements of proteins at the water/methylene chloride interface [11]. The instability of proteins would reduce their bioactivity and potentially cause serious immunoreactions [12]. It has been shown that glucose oxidase (GOD) loses 28% of its activity during the water/oil emulsion process [13]. Therefore, it is necessary to maintain the stability of protein drugs during the process of emulsion and surface loading. 

In aqueous solutions, water molecules can attach to the protein surface to form a hydration shell [14]. In a mixture of water and organic solvent, however, the organic solvent molecules tend to displace the water molecules, both from the hydration shell and from the interior of the protein, distorting the interaction responsible for maintaining the native conformation of the protein [15]. Therefore, the principle for maintaining the stability of a protein is in keeping the surface hydration shell and fixing the conformation of the protein. 

The most widely employed strategy for stabilization of proteins during the emulsion process is the use of additives, such as sugars [16], certain salts [17], amino acids [18], other proteins [19], and polyols [20]. In the presence of these additives, they preferentially interact with the organic solvent to protect proteins. That is the so-called preferential hydration of proteins [21]. However, the protection efficiency of additives depends on their types and concentration, and excess additives would result in the in vivo side effects. For example, Trehalose and mannitol have a significant effect on the recovery of soluble non-aggregated interferon-γ (INF-γ) and growth hormone (GH) after emulsification and ultrasonication [22]; whereas, no or very little protecting effect on insulin-like growth factor-I (IGF-I) is observed [19]. In addition, surface modifications, such as PEGylation, enhances protein stability [23,24]. The water-binding capacity of modifying agents helps to keep a layer of water around the modified protein. However, as the modification is often random, surface chemical modification potentially damages the bioactivity of proteins [25]. Hence, a broad-spectrum method that follows the principle of maintaining the stability of proteins and simultaneously protects their function should be exploited. 

Nanogels are nanometer-scale nanoparticles with polymeric networks that swell in a good solvent [26,27]. Compared with conventional nanocarriers, such as nanospheres [28], nanogels contain a large amount of water in the swollen state to exhibit chaperone-like activity by trapping proteins inside their polymer network. These characteristics endow nanogels with the potential ability of protein protection against organic solvents. To the best of our knowledge, however, there are no studies taking advantage of nanogels and organic coatings to load protein drugs on scaffolds. 

**Scheme 1:**
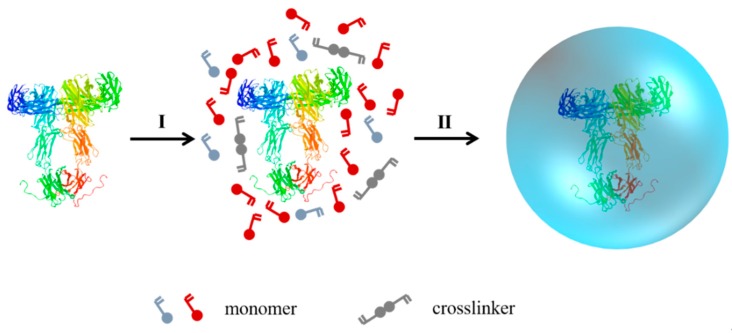
The preparation mechanism of nanogel. Step I: monomers and crosslinkers were enriched around individual protein molecules by electrostatic and hydrogen bond interactions; step II: free radical polymerization was initiated to form a thin polymeric shell around the protein.

Here a kind of chemically cross-linked nanogel is developed to maintain the stability of proteins during the emulsion and scaffold surface loading process, as physically cross-linked nanogels are often unstable in organic solvents [29]. The preparation mechanism of the nanogel is as shown in Scheme 1. When polymeric monomers, such as acrylamide (AAM), acrylic acid (AAC) and 2-methacryloxyloxyethyl phosphorylcholine (MPC), and crosslinkers are added to the protein solution, they can be enriched around individual protein molecules by electrostatic and hydrogen bond interaction. Then in situ free radical polymerization can be initiated by the introduction of ammonium persulphate (APS) and *N*,*N*,*N*’,*N*’-tetramethylethylenediamine (TEMED). Finally, protein molecules can be capsulated by a thin polymeric shell. 

Catalase is used as the model protein to evaluate the protection efficiency of nanogels. Nanogel using AAM or MPC as monomers is denoted as PAAM-n(catalase) or PMPC-n(catalase). The results demonstrate that the preparation process does not affect the stability and activity of proteins. In water-organic solvent mixtures, nanogels can maintain the structure of proteins to keep their activity. Furthermore, the polymeric shell is more hydrophilic, and the protection efficiency of nanogels is higher, implying that nanogels can potentially keep the activity of proteins by maintaining their surface hydration shell. In addition, nanogels can be dispersed in PLA solution and subsequently coated on scaffolds to load catalase with high activity. The exploitation of this kind of nanogels could dramatically expand the application scope of protein drugs and improve their delivery efficiency.

## 2. Materials and Methods 

### 2.1. Chemicals

All chemical reagents used were of analytical reagent grade and purchased from Sigma (Beijing, China) unless otherwise indicated. Catalase and hydrogen peroxide (H_2_O_2_) solution (30%) were purchased from Macklin Biochemical Co., Ltd (Shanghai, China). Acetone, ethyl acetate, and dichloromethane were purchased from Zhengye chemical reagent Co., Ltd (Qingdao, China). PLA (Mw = 5 × 10^6^) was purchased from Daigang Co., Ltd (Jinan, China). The reactive oxygen species (ROS) assay kit was purchased from Solarbio Science & Technology Co., Ltd (Beijing, China). 

### 2.2. Synthesis and Characteristic of PAAM-n(catalase)

Catalase was solved using 20 mM phosphate buffers (PBS) under an ice-bath. AAM and crosslinkers (*N*,*N*’-methylene-bis(acrylamide), BIS) were added sequentially to the catalase solution with stirring. Free-radical polymerization was initiated by adding APS and TEMED into the reaction solution. The final concentration of catalase was adjusted to 1 mg/mL with PBS. The polymerization was allowed to proceed for 120 min in a nitrogen atmosphere at 4 °C. Synthesized nanogels were purified by passing through a hydrophobic interaction column (Phenyl-Sepharose CL-4B) and dialyzed against PBS. The preparation of PMPC-n(catalase) was similar to the above method except that AAM was replaced by MPC. 

The size and zeta potential of PAAM-n(catalase) were measured using dynamic light scattering (DLS, BI-90Plus, Brookhaven Instruments Ltd., USA), and the samples were analyzed in triplicate. Morphology was assessed using a high-resolution transmission electron microscope (TEM, Jem-2100F, JEOL Ltd., Tokyo, Japan), and samples were stained with 2% phosphotungstic acid before observations. 

### 2.3. The Measurement of the Catalase Activity

The activity of catalase and PAAM-n(catalase) was measured by UV-vis absorption spectroscopy. Briefly, native catalase or PAAM-n(catalase) was respectively added to 3 mL of H_2_O_2_ solution (0.1 mol/L), and the final concentration of catalase was 5 μg/mL or 20 μg/mL. The absorbance at 240 nm was measured every 5 seconds for 30 s. The degradation kinetics of H_2_O_2_ was used to indicate the activity of catalase.

### 2.4. The Activity of Catalase treated with Acetone

Native catalase or PAAM-n(catalase) solution (1 mg/mL) was added to acetone, and the volume ratio of the sample solution to acetone was 1:10. Then the mixing solution was stirred at 4 °C for 30 min. After the treatment, a certain amount of mixing solution was added to 3 mL of H_2_O_2_ solution to a final concentration of native catalase or PAAM-n(catalase) of 20 μg/mL. The absorbance at 240 nm was measured every 5 s for 30 s. The degradation kinetics of H_2_O_2_ were used to indicate the activity of catalase.

### 2.5. Characteristic of Catalase treated with Acetone

To test the structural integrity of catalase treated with acetone, the primary and secondary structures of treated catalase or PAAM-n(catalase) were characterized. Native catalase, PAAM-n(catalase), catalase treated with acetone, and PAAM-n(catalase) treated with acetone were heated for 5 min at 95 °C and analyzed via sodium dodecyl sulfate-polyacrylamide gel electrophoresis (SDS-PAGE). Gel electrophoresis was conducted for 2 h on 10% acrylamide gels with a 5% stacking gel at 120 V. The gels were run until the tracking dye had just exited the gel and then were stained with Coomassie blue brilliant R-250. In addition, circular dichroism (CD) analysis was performed on a spectrophotometer (Model J-810, Jasco, Tokyo, Japan). Native catalase, catalase treated with acetone and PAAM-n(catalase) treated with acetone were prepared at 0.2 mg/mL. Measurements were collected at 37 °C over the wavelength range of 250–190 nm.

The morphologies of catalase treated with acetone and PAAM-n(catalase) treated with acetone were assessed using a high-resolution transmission electron microscope (TEM, Jem-2100F, JEOL Ltd., Tokyo, Japan), and samples were stained with 2% phosphotungstic acid before observations.

### 2.6. The Activity of Catalase treated with Ethyl Acetate and Dichloromethane

Native catalase or PAAM-n(catalase) solution (1mg/mL) was added to ethyl acetate or dichloromethane, respectively, and the volume ratios of the sample solution to ethyl acetate or dichloromethane were 1:10. Then, the mixing solution was stirred at 4 °C for 30 min. After the treatment, a certain amount of mixing solution was added to 3 mL of H_2_O_2_ solution and the final concentration of native catalase or PAAM-n(catalase) was 20 μg/mL. The absorbance at 240 nm was measured every 5 s for 30 s. The degradation kinetics of H_2_O_2_ were used to indicate the activity of catalase.

### 2.7. The Hydrophilicity of PAAM-n(catalase) and PMPC-n(catalase)

Native catalase, PAAM-n(catalase) and PMPC-n(catalase) solution were respectively freeze-dried to obtain powder. The resulting powder was pressed into a film and the static contact angle was measured using an automatic contact angle meter apparatus (Kyowa Interface Science Co., Tokyo, Japan) at room temperature.

### 2.8. The Preparation of catalase/PLA-coated titanium dioxide plate 

Catalase or PAAM-n(catalase) was dispersed into the PLA/acetone solution to load onto a titanium dioxide plate. Briefly, native catalase or n(catalase) solution (1 mg/mL, 200 μL) was mixed with PLA/acetone solution (0.5%, *w*/*v*) with volume ratio of 1:1. The mixing solution was stirred at 4 °C for 2 h. Then the solution was uniformly dropped on a titanium dioxide plate (2 cm × 2 cm) and dried at room temperature.

To characterize the catalase/PLA-coated titanium dioxide plate, the surface morphology was assessed using a scanning electron microscopy (SEM, Jsm-7800F, JEOL Ltd., Tokyo, Japan).

### 2.9. The Release of Catalase from the PLA-Coated Titanium Dioxide Plate

To test the release kinetics of native catalase or PAAM-n(catalase), PLA-coated titanium dioxide plate was placed in PBS solution (pH 7.4). The soak solution was changed every 5 days with fresh PBS for 2 months. The concentration of native catalase or PAAM-n(catalase) was measured by an ELISA assay. In addition, the activity of released native catalase or PAAM-n(catalase) was also tested in accordance with the above-mentioned method.

### 2.10. The Determination of Intracellular ROS Concentration

The intracellular ROS concentrations were assessed using an ROS assay kit. In brief, 4T1 cells (4 × 10^5^) were grown on the glass coverslips of a six-well plate, and were incubated with serum-free medium containing 10 μmol/L of a 2’,7’-dichlorodihydrofluorescin diacetate (DCFH-DA) probe for 30 min. At the end of this period, the medium was removed and cells were rinsed three times with PBS. Then, the cells were incubated with complete medium containing H_2_O_2_, catalase/PLA-coated titanium dioxide plate/H_2_O_2_, and PAAM-n(catalase)/PLA-coated titanium dioxide plate/H_2_O_2_. The initial concentration of H_2_O_2_ was ~10 μM and cells were further incubated for 30 min. After the treatment, the fluorescence signal was visualized using confocal microscopy with identical settings for each confocal study. 

## 3. Results and Discussion

### 3.1. The Characteristics of Nanogels 

**Figure 1:**
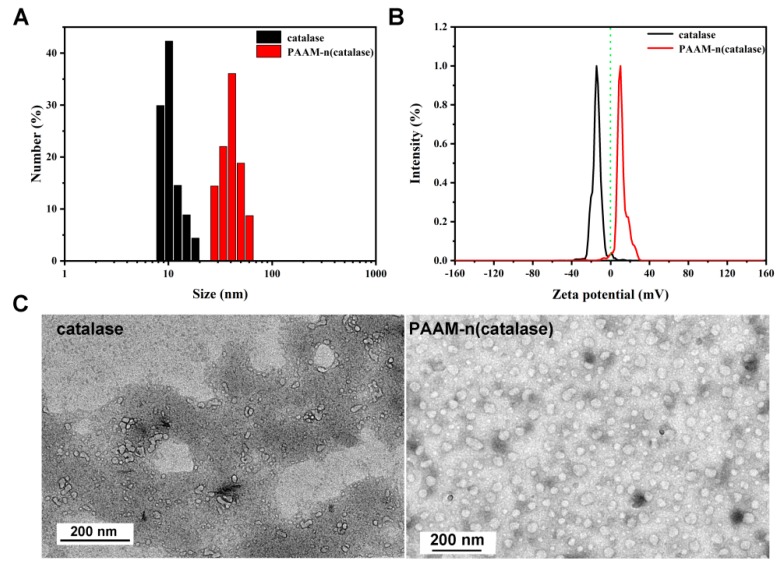
(**A**) The size distribution of native catalase and PAAM-n(catalase); (**B**) the zeta potential of native catalase and PAAM-n(catalase); (**C**) the representative TEM images of native catalase and PAAM-n(catalase).

The preparation of PAAM-n(catalase) was characterized first. AAM was chosen as the model monomer, and *N*-(3-aminopropyl) methacrylamide (APM) was introduced to the polymeric shell to adjust the surface zeta potential of the nanogels. The mean diameter of PAAM-n(catalase) was ~35 nm which was much higher than that of native catalase (~10 nm) [30] (Figure 1A). The zeta potential of native catalase in PBS solution was negative (~−14 mV) as the isoelectric point was at ~pH 5.4 [31]. Due to the shielding effect of the polymeric shell and the existence of poly(APM), the zeta potential of PAAM-n(catalase) was positive (~10 mV). The zeta potential of PAAM-n(catalase) could be adjusted to neutral by decreasing the proportion of APM in the polymeric shell. However, the positive zeta potential could endow nanogels with high stability due to the electrical opposition. Figure 1C shows the representative TEM images of native catalase and PAAM-n(catalase). 

The nanogels showed a spherical morphology with a diameter of 20–40 nm, which is consistent with the DLS measurement. In addition, compared with native catalase, the dispersibility of PAAM-n(catalase) was improved. During the process of TEM sample preparation, many elements, such as high salinity and the drying process, would potentially damage the structure of catalase resulting in their aggregation [32]. The improvement of PAAM-n(catalase) dispersibility implied the protection effect of nanogels on proteins.

### 3.2. The Activity of Native Catalase and PAAM-n(catalase)

**Figure 2:**
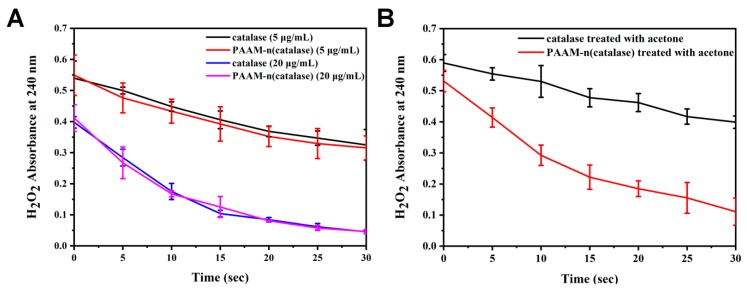
(**A**) The degradation kinetics of H_2_O_2_ which were respectively treated with native catalase and PAAM-n(catalase); (**B**) native catalase and PAAM-n(catalase) were treated with acetone and their activities were tested by the degradation kinetics of H_2_O_2_.

To test the influence of the preparation process on the activity of catalase, the degradation kinetics of H_2_O_2_ were quantified. In the presence of catalase, the concentration of H_2_O_2_ was quickly decreased (Figure 2A), and the reduction rate was dependent on the amount of catalase. PAAM-n(catalase) possessed similar degradation efficiency on H_2_O_2_, demonstrating the activity of proteins was well maintained during the preparation of nanogels. The mild conditions of nanogels formation, such as low temperature, were the main reason for their low damage. 

The protection of catalase against organic solvents by nanogels was also measured. At 20 μg/mL, catalase treated with acetone had a lower catalytic capacity of H_2_O_2_ compared with the native catalase (Figure 2B), while PAAM-n(catalase) treated with acetone did not show an obvious reduction of activity. This result indicated that nanogels could protect proteins from organic solvent damage. 

Actually, many kinds of nanoparticles, such as liposomes [33] and inorganic nanoparticles [34], have been exploited for the entrapment of protein drugs in the past several decades. However, the majority of these nanoparticles cannot protect protein drugs against organic solvents. For example, liposomes may be disintegrated as the organic solvent can interact with the hydrophilic-hydrophobic bilayer [35]. In addition, most of the inorganic nanoparticles load protein drugs through their porosity characteristics [36]. 

An organic solvent can also enter into these holes resulting in the inactivation of protein drugs. Compared with these nanoparticles, chemically cross-linked nanogels possessing high stability can work as the molecular chaperones to protect protein drugs. In addition, we have proven that proteins are not modified during polymerization. As an example, bone morphogenetic protein-2 (BMP-2) can be released from nanogels without any damage [4]. Therefore, this kind of nanogels is an excellent alternative for protein encapsulation. 

### 3.3. The Protection Mechanism of Nanogels 

**Figure 3:**
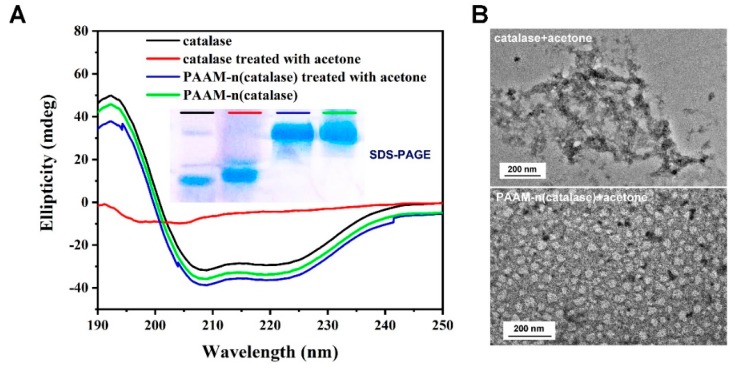
(**A**) Circular dichroism (CD) analysis of native catalase, PAAM-n(catalase), acetone-treated catalase and acetone-treated PAAM-n(catalase); the inset picture shows the SDS-PAGE analysis; (**B**) the representative TEM images of acetone-treated catalase and acetone-treated PAAM-n(catalase).

To explore the protection mechanism of nanogels, the structure change of catalase was tested. The result of the SDS-PAGE analysis showed that the molecular weight of catalase treated with acetone was similar to that of native catalase (inset picture in Figure 3A). This indicated that the organic solvent did not damage the primary structure of proteins. Due to the introduction of the polymeric shells, nanogels had a much higher molecular weight than native catalase. Figure 3A showed the result of circular dichroism (CD) of the proteins. The secondary structure of catalase treated with acetone was destroyed. On the contrary, the secondary structure could be well stabilized by nanogels. 

These results indicated that nanogels fixed the structure of protein leading to the maintenance of their activity. The results of TEM observation further supported this conclusion (Figure 3B). Compared with native catalase, catalase treated with acetone showed obvious aggregation that was often irreversible [37]. This may be because acetone damaged the structure of catalase resulting in the exposure of hydrophobic areas. PAAM-n(catalase) treated with acetone still maintained the spherical morphology, as nanogels could efficiently protect catalase against organic solvents. 

### 3.4. The Protective Effects of Nanogels

**Figure 4:**
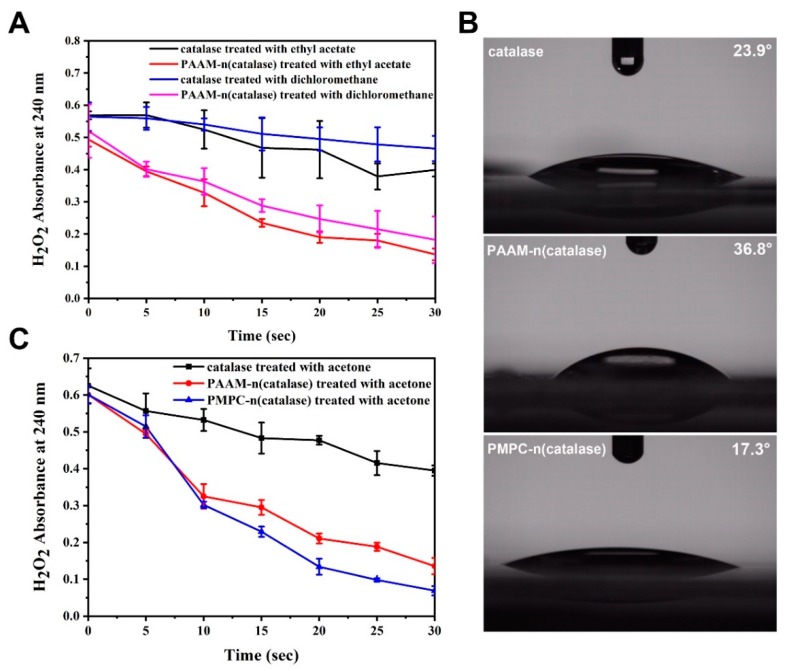
(**A**) Native catalase and PAAM-n(catalase) were treated with organic solvents and their activities were tested by the degradation kinetics of H_2_O_2_; (**B**) native catalase, PAAM-n(catalase), and PMPC-n(catalase) were pressed as films and their contact angles were tested; the magnification was 5 times; (**C**) native catalase, PAAM-n(catalase), and PMPC-n(catalase) were treated with acetone and their activities were tested by the degradation kinetics of H_2_O_2_.

To further prove the protective effects of nanogels, native catalase and PAAM-n(catalase) were treated with different organic solvents and their activities were tested. As shown in Figure 4A, catalase treated with ethyl acetate or dichloromethane had low activity. On the contrary, PAAM-n(catalase) treated with ethyl acetate or dichloromethane could still efficiently degrade H_2_O_2_. It should be noted that dichloromethane caused greater damage to both native catalase and PAAM-n(catalase) than ethyl acetate. This may be because once dichloromethane molecules were bound to the hydrophobic part of a protein chain, they were harder to dissociate. Therefore, we deduced that the protection efficiency of nanogels could be adjusted by changing the surface hydration shell. 

MPC was a type of biomimetic molecules with a super-hydrophilic ability [38]. We used MPC as the polymeric monomer to prepare PMPC-n(catalase). Then native catalase, PAAM-n(catalase), and PMPC-n(catalase) were pressed into films. The contact angle of PMPC-n(catalase) film was 17.3° which was much lower than that of PAAM-n(catalase) film (36.8°) (Figure 4B). This result implied that PMPC-n(catalase) potentially had a more complete hydration shell. The activity test showed that PMPC-n(catalase) treated with acetone had the most rapid degradation rate of H_2_O_2_ (Figure 4C), thus indicating that the PMPC polymeric shell showed better protection against organic solvents than the PAAM polymeric shell. These results indicated that nanogels take advantage of their hydrous polymeric shell to protect proteins against organic solvents.

However, it should be particularly emphasized that the encapsulation efficiency of protein drugs with MPC was lower than that with AAM. Through the protein concentration test, we quantified the encapsulation efficiency of catalase with AAM was at ~85%, while the encapsulation efficiency with MPC was only ~30%. The super-hydrophilicity of MPC endowed nanogels with excellent protein protection ability. The super-hydrophilicity, however, also hindered the enrichment of MPC around individual protein molecules as they possessed a strong interaction with water molecules, such as the strong hydrogen bonds [39]. Therefore, we still chose AAM as the optimal monomer in view of the encapsulation efficiency and protection ability.

### 3.5. The Characteristics of Nanogels Loaded on Titanium Dioxide Plate by PLA Coating 

**Figure 5:**
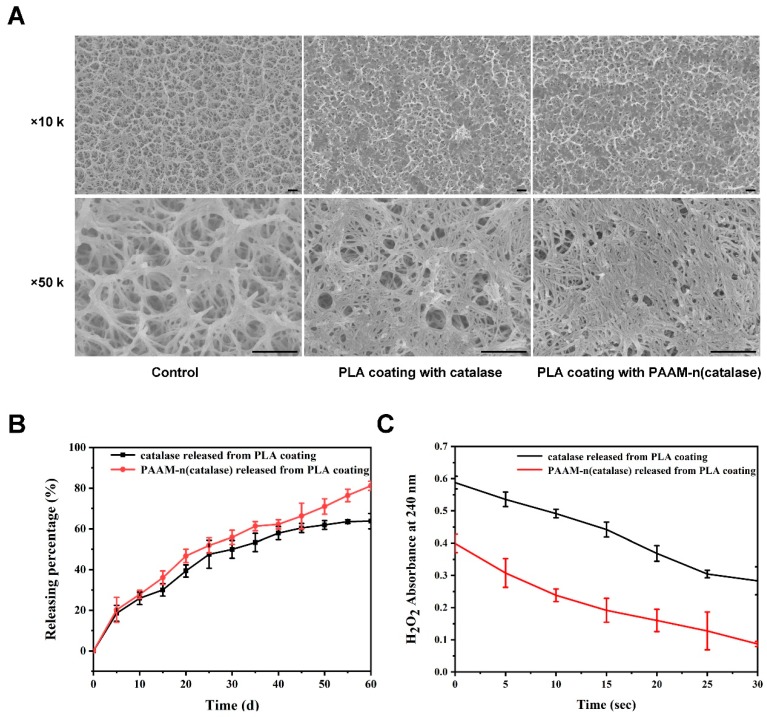
(**A**) The representative SEM images of a titanium dioxide plate and a PLA-coated titanium dioxide plate; the bar was 500 nm; (**B**) the release profiles of native catalase and PAAM-n(catalase) from the PLA coating; (**C**) the activity of released catalase was tested by the degradation kinetics of H_2_O_2_.

We further loaded native catalase and PAAM-(catalase) on a titanium dioxide plate using PLA coating. The representative SEM images are shown in Figure 5A. The surface holes of the titanium dioxide plate became smaller with the coating of PLA. The release kinetics of native catalase and PAAM-(catalase) from a PLA coating were also tested. As shown in Figure 5B, a burst release phase within the first few days was observed. This might be due to the initial release being diffusion-controlled [40]. After 60 days, ~80% of PAAM-(catalase) was shown to be released from the PLA coating, while ~60% of native catalase was released. The delayed release of native catalase might be associated with the non-specific adsorption to the PLA matrix or non-covalent aggregation of catalase [40]. 

Nanogels could reduce the potential adsorption or aggregation of proteins, facilitating their efficient release. In addition, nanogels efficiently maintained the activity of released proteins. Compared with native catalase released from a PLA coating, PAAM-n(catalase) released from a PLA coating showed a faster H_2_O_2_ degradation rate. On one hand, the organic solvent could damage the proteins during the loading process. On the other hand, protein instability during release should be attributed to a local pH drop, as the degradation of the PLA results in the acidification of the medium [41]. These results demonstrated that nanogels could efficiently protect proteins when they were loaded into the organic coating and subsequently released from it.

### 3.6. The Detection of the ROS Concentration in 4T1 Cells 

**Figure 6:**
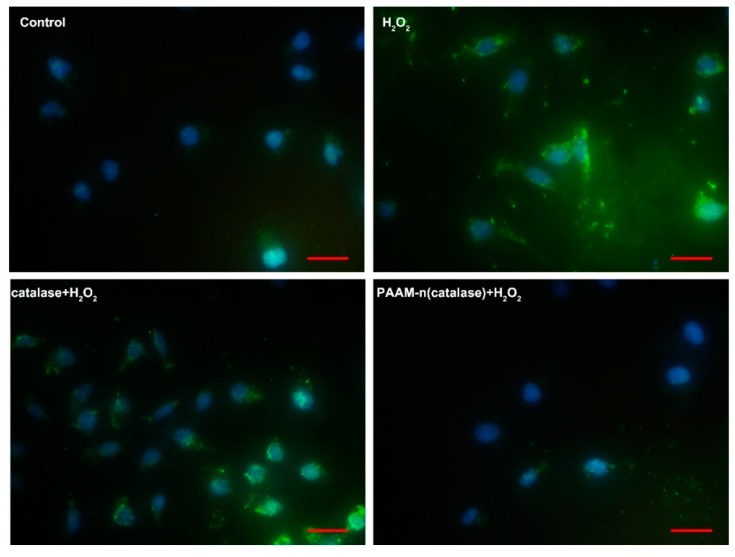
The reactive oxygen species (ROS) concentration in 4T1 cells that were treated with H_2_O_2_, catalase/PLA-coated titanium dioxide plate/H_2_O_2_, and PAAM-n(catalase)/PLA-coated titanium dioxide plate/H_2_O_2_. The bar was 20 μm.

To further prove the activity of native catalase and PAAM-n(catalase) loaded on a titanium dioxide plate with PLA coating, we detected the ROS concentration in 4T1 cells, which were respectively co-cultured with H_2_O_2_ and a PLA-coated titanium dioxide plate. As shown in Figure 6, normal 4T1 cells contained a small amount of ROS, which was quantified with green fluorescence. When treated with H_2_O_2_, the ROS concentration in live cells was dramatically increased, proving that extracellular H_2_O_2_ had an effect on intracellular ROS concentration. Native catalase-loaded titanium dioxide plate could partially degrade H_2_O_2_, decreasing the ROS concentration in live cells to a certain extent. On the contrary, a PAAM-n(catalase)-loaded titanium dioxide plate could efficiently reduce the in vivo ROS content. These results further verified the protection ability of nanogels.

In this study, an enzyme was chosen as the model protein drug. Hence, we exploited a kind of non-degradable nanogel as substrates of enzymes are often small molecules. The protection ability of nanogels was mainly dependent on the polymeric shells formed by monomers [42,43]. Therefore, through changing the crosslinkers, we could also design the responsive-release nanogels with high protection ability to load other functional protein drugs, such as monoclonal antibodies [44] and growth factors [4], on scaffolds with organic coatings. In other words, we exploited a platform for the loading of protein drugs on scaffolds using organic coatings.

## 4. Conclusions

We developed a type of chemically cross-linked nanogel to maintain the stability of protein drugs. These nanogels take advantage of their hydrous polymeric shell to protect protein drugs against organic solvents. Furthermore, nanogels could efficiently protect proteins when they were loaded on scaffolds using organic coatings and released from them. To the best of our knowledge, this is the first use of hydrophilic nanogels as a protection niche to load protein drugs on scaffolds through an organic coating. The exploitation of this kind of nanogels could dramatically expand the application scope of protein drugs and improve their delivery efficiency.
